# Supplementary material for: Antitumor Activity of Simvastatin in Preclinical Models of Mantle Cell Lymphoma
Source: Cancers (Basel). 2022 Nov 15;14(22):5601. doi: 10.3390/cancers14225601 (PMC9688202; doi:10.3390/cancers14225601)
Supplement: Supplementary file 1 [file cancers-14-05601-s001.zip › cancers-1968810-supplementary.pdf]

Supplementary Materials: Antitumor Activity of Simvastatin in Preclinical Models of Mantle Cell Lymphoma

Juliana Carvalho Santos, Núria Profitós-Pelejà, Marcelo Lima Ribeiro and Gaël Roué

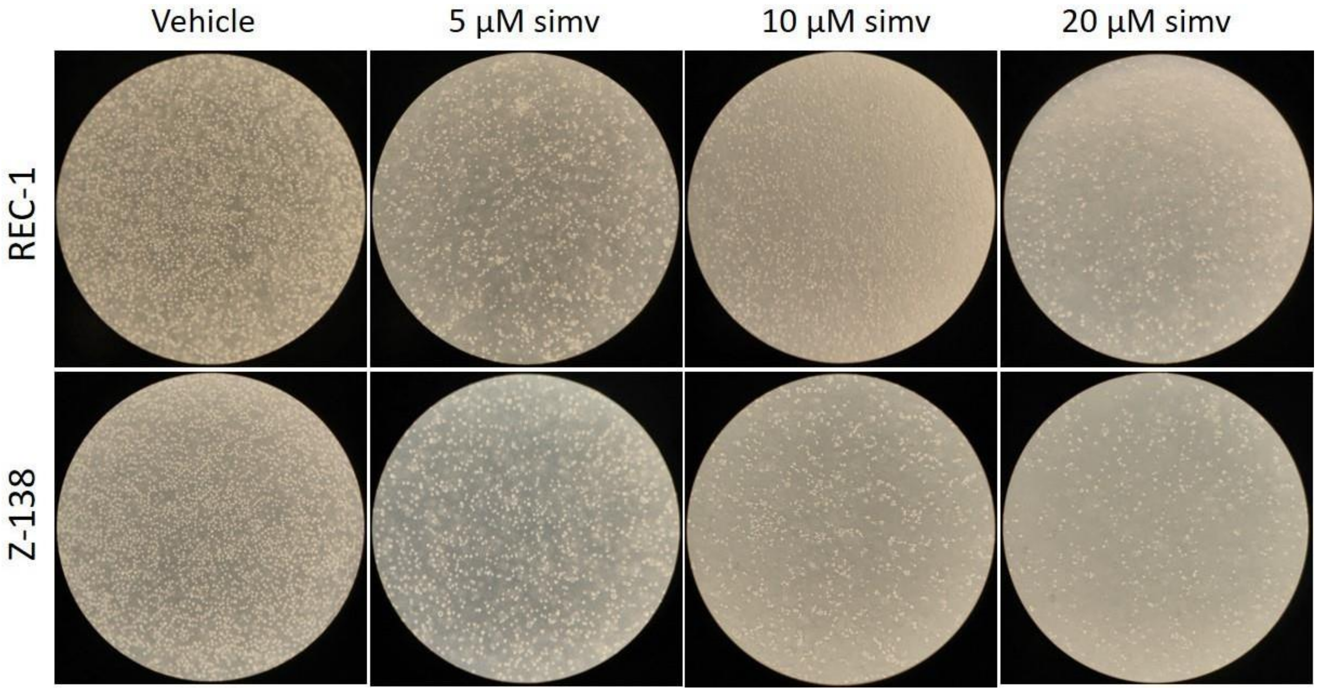

Figure S1. Representative bright field pictures of REC-1 and Z-138 cultures exposed for 3 days to increasing doses of simvastatin.

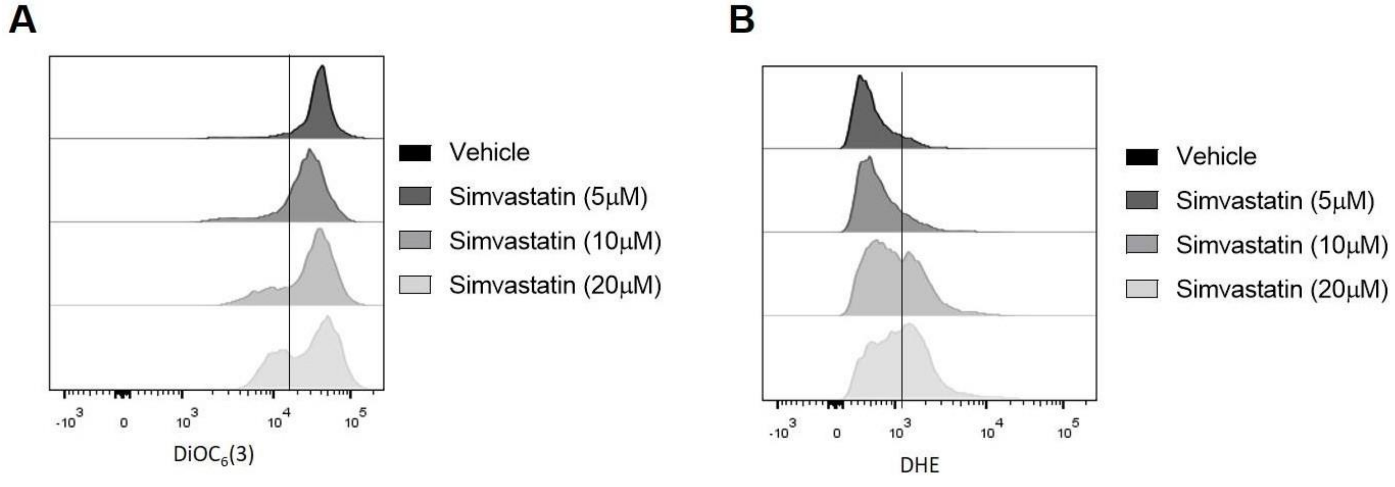

Figure S2. Representative FACS images of REC-1 and Z-138 cell lines treated with simvastatin or vehicle for 48 h analyzed by DiOC6 (A) and dihydroethidium (B) staining.

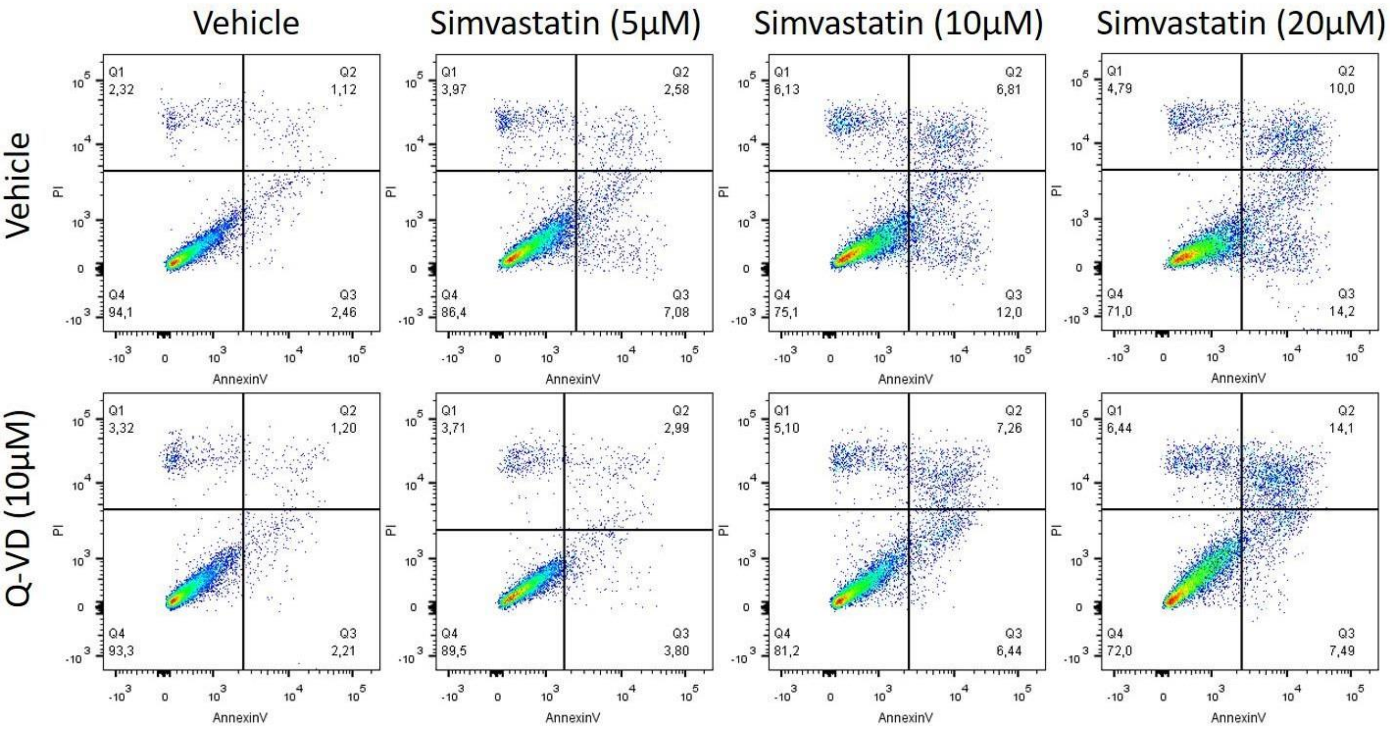

Figure S3. Representative FACS images of REC-1 and Z-138 cell lines treated with simvastatin or vehicle for 48 h analyzed by Annexin V-FITC/propidium iodide staining.

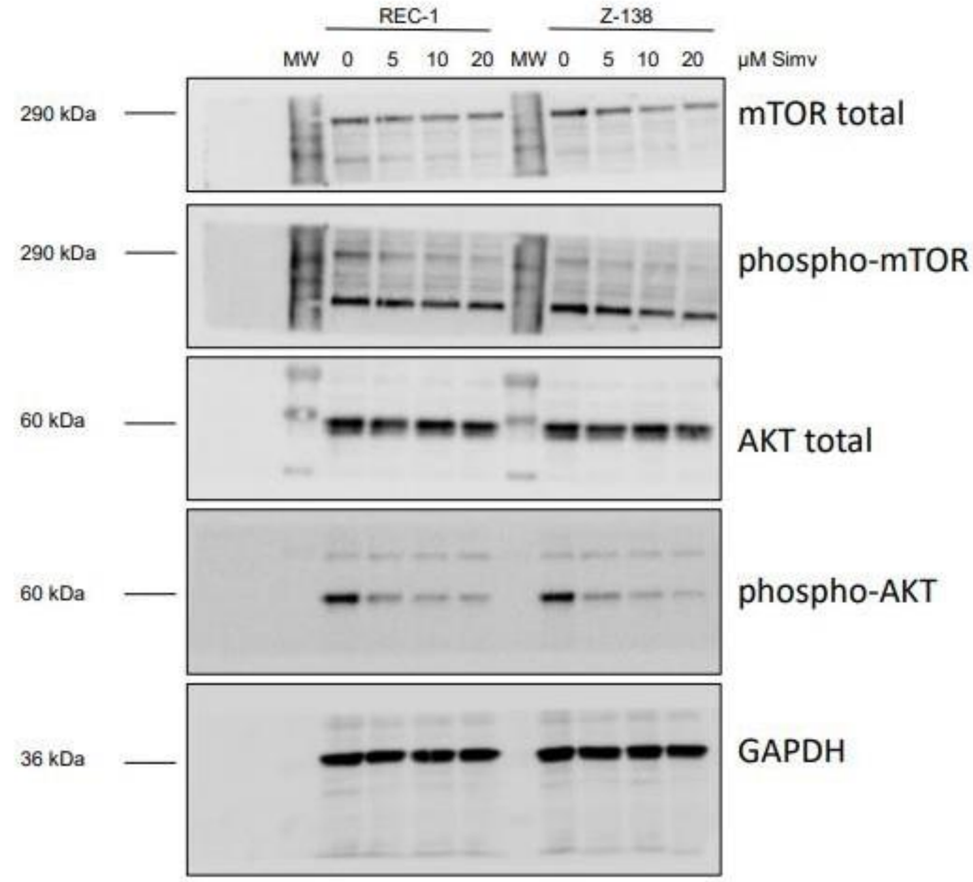

Figure S4. Full Western blot images.
